# A Complex mHealth Coaching Intervention to Prevent Overweight, Obesity, and Diabetes in High-Risk Women in Antenatal Care: Protocol for a Hybrid Type 2 Effectiveness-Implementation Study

**DOI:** 10.2196/51431

**Published:** 2023-09-18

**Authors:** Sharleen L O'Reilly, Rachel Laws, Helle Terkildsen Maindal, Helena Teede, Cheryce Harrison, Fionnuala M McAuliffe, Aisling Geraghty, Cristina Campoy, Mercedes G Bermúdez, Laura Pirhonen, Christy Burden, Anna Davies, Ditte Hjorth Laursen, Timothy Skinner, IMPACT DIABETES B2B Consortium

**Affiliations:** 1 School of Agriculture and Food Science University College Dublin Dublin Ireland; 2 University College Dublin Perinatal Research Centre, School of Medicine University College Dublin National Maternity Hospital Dublin Ireland; 3 School of Exercise and Nutrition Science Deakin University Burwood Australia; 4 Department of Public Health Aarhus University Aarhus Denmark; 5 Monash Centre for Health Research and Implementation Monash University Melbourne Australia; 6 Department of Paediatrics School of Medicine, EURISTIKOS Excellence Centre for Paediatric Research University of Granada Granada Spain; 7 Instituto de Investigación Biosanitaria Health Sciences Technological Park Granada Spain; 8 Institute of Psychology University of Copenhagen Copenhagen Denmark; 9 Bristol Medical School University of Bristol Bristol United Kingdom; 10 Liva Healthcare Copenhagen Denmark; 11 Australian Centre for Behavioural Research in Diabetes Deakin University Geelong Australia; 12 See Acknowledgements

**Keywords:** hybrid type 2 effectiveness-implementation, gestational diabetes, obesity, mHealth, mobile health, health behavior change, pregnancy, postpartum, weight management, health coaching, maternal health

## Abstract

**Background:**

Women with overweight and obesity are at higher risk of developing complications in pregnancy such as gestational diabetes and longer-term chronic conditions. Research concerning health behavior change interventions during pregnancy and postpartum shows promising effects, but implementation into routine services is sparsely investigated. Most interventions focus on the antenatal or postpartum life stages, failing to meet the needs of women. IMPACT DIABETES Bump2Baby is a multicenter project across 4 high-income countries developed to test the implementation of an antenatal and postpartum evidence-based mobile health (mHealth) coaching intervention called Bump2Baby and Me (B2B&Me) designed to sit alongside usual care in the perinatal period.

**Objective:**

We aim to explore the feasibility and implementation of the B2B&Me intervention and investigate the effectiveness of this intervention in women at risk of gestational diabetes.

**Methods:**

IMPACT DIABETES Bump2Baby is a hybrid type 2 effectiveness-implementation study, which integrates an evidence-based mHealth coaching app that includes personalized health behavior change coaching provided by health care professionals alongside antenatal care from the first antenatal visit to 12 months postpartum. The mHealth app offers the possibility of synchronous calls, asynchronous contact (including coach-participant text and video messaging exchanges tailored to the participant’s needs), and ongoing access to an extensive library of bespoke intervention materials. Participants will interact asynchronously with their health coach throughout the intervention via the app. This randomized controlled trial across 4 clinical sites within Ireland, the United Kingdom, Spain, and Australia will recruit 800 women in early pregnancy to evaluate the effectiveness on postpartum weight. The Exploration, Preparation, Implementation, and Sustainment implementation framework is the theoretical underpinning of the study. The implementation evaluation will be assessed at the individual, hospital staff, and broader community levels using the Reach, Effectiveness, Adoption, Implementation, Maintenance (RE-AIM) framework. Data sources for the RE-AIM evaluation will include app and platform analytics, screening and training records, participant medical records, key informant interviews, participant and partner exit interviews, cost data, study questionnaires, staff surveys, and blood sample analyses.

**Results:**

The study was approved and registered with the Australian New Zealand Clinical Trials Registry on November 19, 2020. Recruitment commenced on February 9, 2021, and data collection is ongoing. Publication of the results is expected in 2024.

**Conclusions:**

This is the first hybrid effectiveness-implementation study of an 18-month mHealth coaching intervention in at-risk women that we are aware of. As research aims to move toward real-world implementable solutions, it is critical that hybrid studies are conducted. The data from this large multicenter study will be useful in planning the potential implementation and scale-up of evidence-based perinatal health behavior change interventions.

**Trial Registration:**

Australian New Zealand Clinical Trials Registry ACTRN12620001240932; https://www.anzctr.org.au/Trial/Registration/TrialReview.aspx?id=380020&isReview=true

**International Registered Report Identifier (IRRID):**

DERR1-10.2196/51431

## Introduction

Overweight and obesity are growing worldwide health concerns. For women in their reproductive years, this brings an increased risk of serious pregnancy complications such as preeclampsia, gestational diabetes mellitus (GDM), cesarean delivery, and stillbirth [1,2]. Obesity in pregnancy impacts offspring health with higher rates of congenital anomaly, large for gestational age, childhood obesity [3-5], gut microbiota dysbiosis, behavioral problems, or altered brain functioning, among others [6]. GDM is one of the most common pregnancy complications impacting approximately 1 in 7 pregnancies worldwide [7], with immediate and long-term health impacts for both mother and baby [8-10]. Excessive gestational weight gain (GWG) increases the risk of developing GDM and independently increases child obesity risk [2,11] and a woman’s risk of obesity, heart disease, and type 2 diabetes (T2DM). GWG is frequently retained after birth and compounded by interpregnancy weight gain with a cycle of increasing weight through the reproductive years [12,13].

Pregnancy is a key time point to intervene and yield a benefit for both mother and child [12]. Meta-analyses show that behavioral interventions during pregnancy provide cost-effective support for optimizing GWG, GDM prevention and treatment, T2DM conversion prevention, and postnatal weight retention [14-16]. Meta-analyses have also identified that antenatal behavioral interventions are associated with reduced GDM and emergency cesarean delivery, and as a result the US Preventative Task Force now recommends implementing them [17]. Interventions to reduce postpartum weight retention are challenging to deliver but offer health benefits [18] with individual participant meta-analysis data showing that decreasing prepregnancy or early pregnancy maternal BMI reduces childhood obesity [11]. Current evidence, therefore, supports the efficacy of antenatal, postpartum, and interpregnancy health behavior change interventions in diet and physical activity during pregnancy and postpartum for improving maternal and child outcomes. Key evidence gaps include that studies typically focus on intervening during either the antenatal or postpartum period but not both and that studies tend to be short in duration (6 months is the average duration), which is known to be less effective at supporting sustained health behavior change [19-21]. Importantly, a large research translation gap exists around achieving adequate population penetration, participation, and implementation at scale with efficacious prevention interventions [19,20,22,23]. Limited studies have explored intervention implementation aspects beyond highly controlled clinical trials [19,23].

While the implementation of health behavior change interventions is recommended at a population level, identifying women at higher risk of adverse outcomes for targeted intervention is likely to yield improved health and economic benefits. This includes identifying women at risk of developing GDM through risk factor screening [24,25] and recognizing higher risks in different BMI groups with variable GWG guidelines [26]. However, few studies have focused on implementing these approaches with large variations in guidelines [27] and limited uptake in practice [8], which fails to identify pregnant women who would benefit from intervention. Postpartum and interpregnancy reach, penetration, and participation are all key barriers to both efficacy and implementation, and evidence is not yet strong enough to recommend population-level scale-up. Few studies have engaged women in pregnancy to optimize reach and participation in the face of competing family demands and less contact with the health system [28]. Again, implementation research in the real world that addresses these barriers is vital to inform population-level strategies and future roll-out and scale-up.

## Methods

### Context

**Figure 1:**
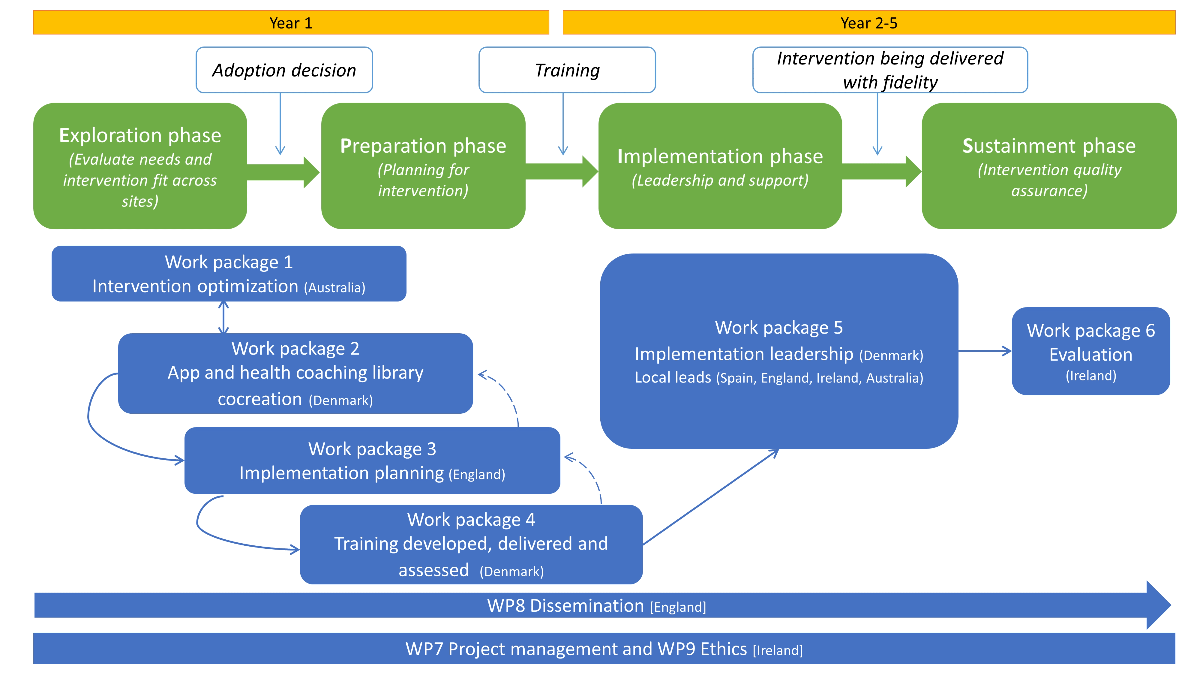
The Exploration, Preparation, Implementation, and Sustainment framework applied to the IMPACT DIABETES Bump2Baby project (2020-2024).

IMPACT DIABETES Bump2Baby (IDB2B) is a 5-year European Union and Australian research innovation project looking to demonstrate the implementation of an evidence-based system of care for the prevention of T2DM, overweight, and obesity in women at risk of GDM (Figure 1). It is designed to be delivered in the antenatal setting of 3 European countries (Ireland, England, and Spain) and Australia. Within IDB2B, the Bump2Baby and Me (B2B&Me) health behavior change intervention was designed during formative work and will be subject to a randomized controlled trial (RCT) to test the integration of screening for at-risk women, the mobile health (mHealth) app, and personalized health coaching in pregnancy through to 12-month postpartum. The detailed B2B&Me RCT protocol is published elsewhere [29], with the focus here being on the implementation protocol. The formative work included the selection of the GDM risk screening tool. The Monash tool [30] was selected as it incorporated overweight and obesity, it was shown to have reasonable accuracy, it was based on the best available evidence, and it was validated internationally [31]. The B2B&Me intervention design was also closely informed by robust evidence from pregnancy including systematic synthesis of the evidence on health behavior change interventions in pregnancy [15,19] and postpartum [18,20,32] to identify key intervention and implementation components required in our intervention study design. The B2B&Me intervention design was also closely informed by robust evidence from pregnancy [33,34] or postpartum [35,36] low-intensity, theoretically underpinned, and low-cost interventions led by the international authorship team. These health behavior change interventions [33-36] were systematically deconstructed and reconfigured considering the systematic synthesis and the Behaviour Change Wheel Framework [37] to develop the B2B&Me intervention. The intervention was designed to be delivered using mHealth, which enables remote delivery via an app and personalized remote health coaching during pregnancy and postpartum, and to run in parallel to standard health service provision. The design of the health coaching via a health care professional and optimized health behavior change content in the app was focused on supporting a meaningful coaching relationship with the woman at the center of B2B&Me.

### Aims

The overarching aim of IDB2B is to contribute to the prevention of maternal and child overweight, obesity, T2DM, and other noncommunicable diseases. It seeks to achieve this through a hybrid type 2 effectiveness-implementation study that combines evidence-based antenatal and postpartum health behavior change interventions into a single seamless support program (B2B&Me) that optimizes health outcomes for the mother and baby and contributes to the prevention of T2DM and adverse long-term programming effects in the offspring. The primary efficacy outcome of the B2B&Me trial will be reduced postpartum weight gain and secondary health outcomes include the health of the mother and child. The secondary implementation aims are to explore the fidelity of health coaching training, delivery, and receipt; the feasibility of the B2B&Me implementation strategy through a process evaluation; and the intervention’s reach, cost-effectiveness, adoption, implementation, and maintenance.

### Study Design

This is a type 2 hybrid effectiveness-implementation study. Type 2 hybrid designs have a “dual focus on the clinical intervention and implementation-related factors” with a clear implementation strategy that is deemed plausible in the real world [38]. Here, implementation factors may be secondary outcomes or coprimary ones [39]. We plan to study the effectiveness of the intervention using the B2B&Me RCT data as the primary outcome. We will evaluate the implementation process using the Reach, Effectiveness, Adoption, Implementation, Maintenance (RE-AIM) framework [40] to examine the reach, efficacy, adoption, implementation, and maintenance as secondary outcomes.

### Health System Context

The health care systems in Ireland, England, Spain, and Australia are similar. They use a government-supported system where universal free care is provided to all eligible citizens and residents. The private health system is funded by individuals and subsidizes the costs of private care and provides greater choice in where health care is provided (public, private, or semiprivate). IDB2B was located within public care systems, and Figure 1 describes the work packages that formed the project.

### Framework

The Exploration, Preparation, Implementation, and Sustainment (EPIS) framework was used to scaffold IDB2B [41,42]. The framework was developed out of the public sector and allied health services systems in America and has 4 main implementation process phases (exploration, preparation, implementation, and sustainment) with outer and inner organizational contexts and factors identified [41]. EPIS also includes innovation factors that relate to newly implemented intervention characteristics and bridging factors that explore the interplay between inner and outer contexts [41]. The exploration phase for IDB2B will involve evaluating current needs to ensure the intervention fits across system levels, organization levels, and staff and recipient levels through key informant interviews, site-level surveys, and systematic reviews. The preparation phase involved planning for the B2B&Me intervention through staff training, usual care service mapping, and awareness raising. The implementation and sustainment phases will be assessed using the RE-AIM framework [40], which measures results along the dimensions of reach, effectiveness, adoption, implementation fidelity, and maintenance.

### Study Sites and Eligibility Criteria

IDB2B delivered the B2B&Me RCT in a single maternity health service site within Ireland, Spain, England, and Australia, and the usual care provided in each one is described in Table 1. The Monash GDM screening tool will be used to screen women at all sites attending antenatal care in their first trimester [30]. Women scoring ≥3 are eligible to enroll. The IDB2B study population will include women of reproductive age 18 years or older and attending their service for maternity care in the study sites that meet the B2B&Me eligibility criteria [29]. Key informant interviews will be conducted with senior staff involved in the delivery of antenatal services at each site. The interviews will also be conducted with the research teams and health coaches involved in delivering B2B&Me at each site. General hospital staff will be approached to participate in site-level surveys. All staff within the sites will be eligible to participate in the site-level surveys and the key informant interviews.

**Table 1:** Description of 2021-2022 usual maternity care across the 4 clinical sites involved in the Bump2Baby and Me randomized controlled trial in women at risk of gestational diabetes (2021-2023).

	National Maternity Hospital, Dublin, Ireland	Southmead Hospital, North Bristol NHS Trust, Bristol, United Kingdom	San Cecilio University Hospital, Granada, Spain	Monash Medical Centre, Monash Health, Melbourne, Australia
Service	Urban; maternity, gynecological, neonatology, and reproductive care provided; Dublin and North Wicklow communities serviced; 194 inpatient beds	Urban; adult care including maternity services provided; Bristol, South Gloucestershire, and North Somerset communities serviced; 996 inpatient beds	Urban; adult care including maternity services provided; Granada communities serviced; 547 inpatient beds	Urban; adult care including maternity services provided; greater Melbourne residents serviced; 640 inpatient beds
Staff, n	930	12,000	3200	4000
Babies delivered, n	7855	6000+	2265	3800
Number diagnosed with GDM^a^, n	774	~600 [43]	68-204	760
Rates of high BMI in pregnancy (≥25 kg/m^2^) (%)	43.5	50.2 [44]	43.2	51 [45]
Notification of pregnancy	Women notify hospital	A community midwife is notified first and then transferred to the hospital	Women notify hospital	Women notify hospital
Models of care available	Midwife-led, medical team-led, or specialist care	Midwife-led, medical team-led, or specialist care	Midwife-led, medical team-led, or specialist care	Midwife, collaborative (midwife and medical team), specialty, shared (affiliated community provider and hospital), or medical team-led specialist care
Universal screening for GDM and test performed	No—HSE^b^ guidelines followed [46], 2-step 50 g GCT^c^ followed by 100 g 3-hour GTT^d^	No—NICE^e^ guidelines followed [47], 75 g 2-hour GTT	Yes—SEGO^f^ and GEDE^g^ guidelines followed [48,49], 100 g 3-hour GTT	Yes—ADIPS^h^ group guidelines followed [50], 75 g 2-hour GTT
GDM diagnostic criteria	Fasting ≥5.3 mmol/L, 1-hour ≥10.0 mmol/L, 2-hour ≥8.6 mmol/L, 3-hour ≥7.8 mmol/L; 2 out of range values needed for diagnosis	Fasting ≥7.0 mmol/L, 2-hour ≥7.8 mmol/L; 1 out of range l value needed for diagnosis	Fasting ≥5.8 mmol/L, 1-hour ≥10.6 mmol/L, 2-hour ≥9.2 mmol/L, 3-hour 8.1 mmol/L; 2 out of range value needed for diagnosis	WHO^i^ (2013) ≥5.1 mmol/L fasting, ≥10.0 mmol/L 1-hour, ≥8.5 mmol/L 2-hour; 1 out of range value needed for diagnosis
Care pathway for high BMI	BMIs >25, access to group healthy eating and physical activity health behavior change education program	Nil	Nil	Limited—women with BMIs >35 may have access to group healthy eating and physical activity health behavior change education program
Care pathway for GDM	A multidisciplinary team provides support with diet and physical activity being the primary intervention followed by metformin or insulin management	A multidisciplinary team provides support with diet and physical activity being the primary intervention followed by medication or insulin management	Multidisciplinary team provides support with diet and physical activity being the primary intervention followed by medication or insulin management	Multidisciplinary team provides support with diet and physical activity being the primary intervention followed by medication or insulin management
Postdischarge care	Public health nurse–delivered, visits recommended—72 hours after discharge, 2 weeks, 6 weeks, 2 months, 3 months, 4 months, 6 months, 9 months, and 12 months	Health visitor or midwife delivered, visits recommended—within 72 hours after birth, 1-2 weeks, 6-8 weeks, and 9-12 months	Maternal and child health nurse delivered, visits at 1-2 weeks and 3 and 12 months after birth	Maternal and child health nurse delivered, visits recommended—home visit after birth, 2 weeks, 4 weeks, 8 weeks, 4 months, 8 months, and 1 year
Postpartum GDM care recommended	General practice normal location for 6- to 12-week postpartum glucose tolerance test, annual diabetes screening with preconception planning [46]	General practice normal location for 6- to 13-week postpartum fasting plasma glucose test or glycated hemoglobin if after 13 weeks postpartum, annual diabetes screening with preconception planning [47]	General practice normal location for 6- to 8-week postpartum oral glucose tolerance test, annual diabetes screening with preconception planning [49]	General practice for their 6- to 12-week postpartum glucose tolerance test and regular diabetes screening is recommended [50]

^a^GDM: gestational diabetes mellitus.

^b^HSE: Health Service Executive.

^c^GCT: glucose challenge test.

^d^GTT: glucose tolerance test.

^e^NICE: National Institute of Clinical Excellence.

^f^SEGO: Sociedad Española de Ginecología y Obstetricia.

^g^GEDE: Grupo Español de Diabetes y Embarazo.

^h^ADIPS: Australian Diabetes In Pregnancy Study.

^i^WHO: World Health Organization.

### Intervention

**Figure 2:**
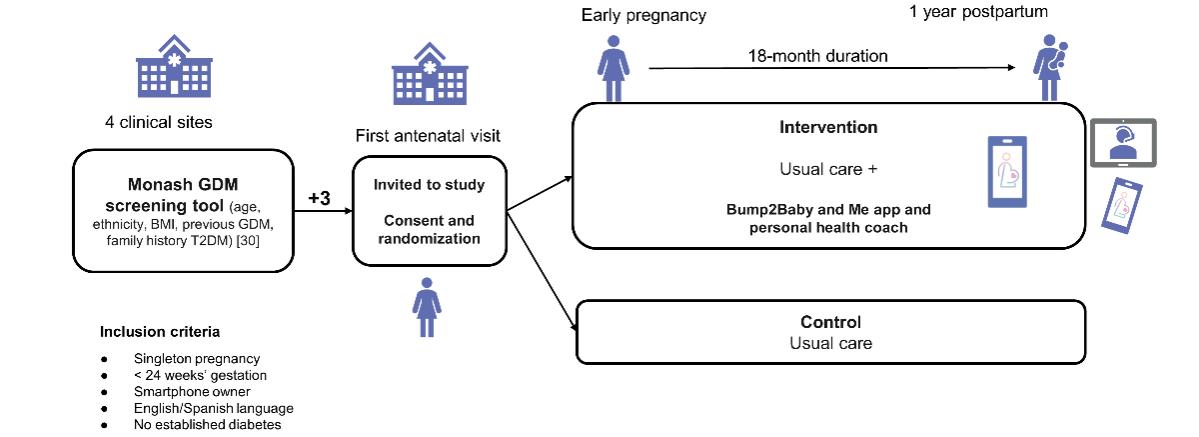
Overview of the Bump2Baby and Me (B2B&Me) randomized controlled trial in women at risk of developing gestational diabetes (2021-2023). GDM: gestational diabetes; T2DM: type 2 diabetes.

**Figure 3:**
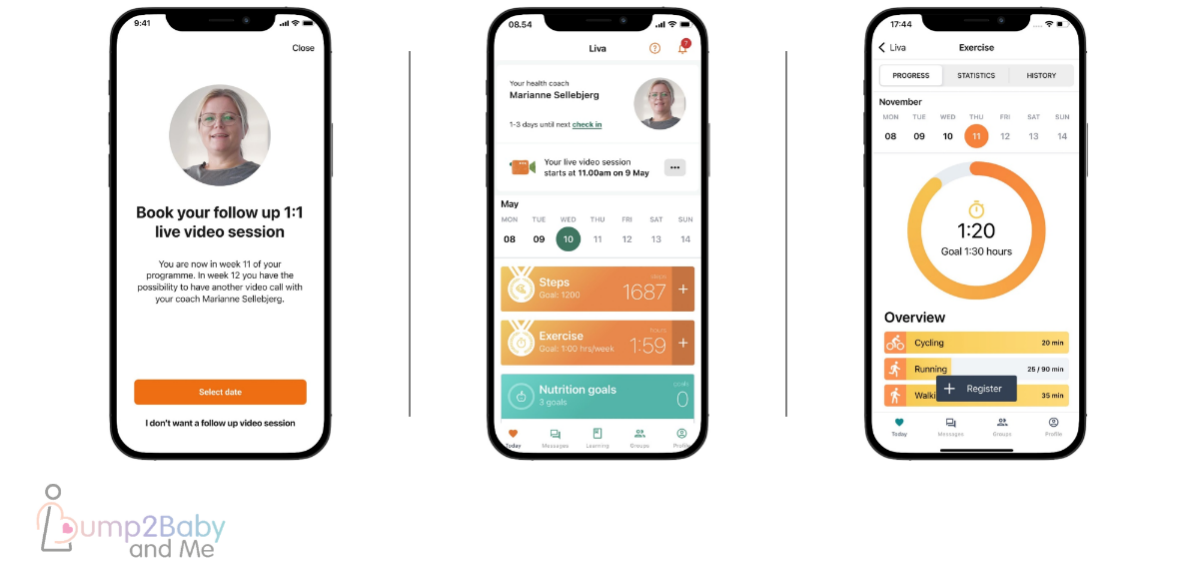
Screenshots of the mobile health coaching app Bump2Baby and Me.

While the intervention is described elsewhere [29], briefly, it consists of a hybrid model that consisted of a smartphone app (Liva Healthcare provider) and linked health care professional health coaching (Figure 2). The health behavior change techniques within the intervention combine 3 frameworks that share a core person-centered philosophy to guide the structure of the program delivery and integration of behavior change techniques and relational techniques, these being social cognitive theory [51], the reciprocal triad of causation [51], self-determination theory [52], and the transtheoretical model of behavior change [53]. The health coaches will be allied health care professionals with an interest or training in midwifery and maternal and child health. They will receive 5 days of training prior to engaging with participants. The health coaching training will be a mixture of pedagogical approaches, including direct instruction, modeling, skills practice, and problem-solving, all supported by self-directed learning and self-reflection, outside of the formal 5-day program. Liva Healthcare has an existing internal quality development and assurance program where team leaders conduct regular supervision meetings with coaches. These sessions are frequent for new coaches until they demonstrate the expected coaching competencies based on systematic observation of coaching data. Hereafter, the frequency decreases over time to quarterly supervision sessions. Women will commence the intervention in their early pregnancy and will be offered a personalized baseline session where they meet their health coach during a synchronous web-based video call lasting 45-60 minutes. During this session, coaches establish a relationship with each woman, support them to set some initial health goals, and instruct them on the use of the smartphone app. The app offers the possibility of synchronous calls, asynchronous contact (which includes text and video messaging exchanges between the coach and participant tailored to the woman’s needs), and ongoing access to an extensive library of materials developed specifically for the intervention (Figure 3). The women will interact asynchronously with their health coach throughout the intervention via the smartphone app. The health coach will have access to a dashboard that allows them to provide asynchronous, continuous coaching across a large volume of women. During pregnancy, the asynchronous interactions occur once a week for the first 4 weeks and then biweekly until birth, resulting in a total of 14 contacts. If the woman is diagnosed with GDM (typically 24-26 weeks of gestation), an additional 15-minute synchronous video call will be included as well as additional educational material on GDM. The health coaching content during pregnancy centers on diet and physical activity for a healthy pregnancy, it also includes emotional aspects, breastfeeding preparation, expectations for delivery, and so forth. For the postpartum period, a synchronous call is scheduled for 6-8 weeks after delivery to re-engage participants in the coaching program, to have a delivery debrief with the coach, and to set new goals tailored to the postpartum period. This is followed by 1 weekly and 22 biweekly asynchronous contacts. The postpartum health coaching content centered on diet and physical activity for the woman and child including infant feeding and active play. Participants can spread out the timing of any contacts to suit their needs. The smartphone app collects detailed analytics on the woman’s time spent in the app, number of resources accessed, type and amount of goals set, goal progress, communications with health coaches (number of coach messages accessed and responded to), communications with peers within the app, and self-monitoring data (steps, activity, and diet).

### Data Collection

#### Overview

Table 2 demonstrates the RE-AIM evaluation framework for the IDB2B project. Each evaluation item is described in greater detail.

**Table 2:** Evaluation framework for IMPACT DIABETES Bump2Baby project (2020-2024) that incorporates the Bump2Baby and Me randomized controlled trial across 4 countries in women at risk of developing gestational diabetes.

RE-AIM^a^ dimension and definition		Indicator	Data sources
**Reach**			
	Penetration and participation in B2B&Me^b^	% women screened and randomized out of hospital population (penetration) % women eligible agreeing to participate in the trial (trial participation)	Screening records in IDB2B^c^ database
		% women completing minimum health coaching sessions (health coaching participation) App and platform analytics for intervention women (mHealth^d^ engagement)	Liva Healthcare app and platform analytics
		Characteristics of participants by each site compared with the hospital population (representativeness)	Screening records in IDB2B database and hospital annual reports
**Effectiveness**			
	B2B&Me impact on outcomes	Change in BMI at 12 months postpartum % women with any complications recorded Change in BMI at delivery % women diagnosed with GDM^e^ % women delivered via each mode of birth Placenta weight recorded (g) Infant birth weight (g), length (cm), and head circumference (cm) Duration of breastfeeding Change in physical activity/diet/sleep/quality of life/depression/blood glucose and lipid levels at 12 months postpartum Infant growth pattern and neurodevelopment during the first year of life	B2B&Me questionnaires and blood sample analysis
		% women scoring ≥3 on Monash Gestational Diabetes Risk Scoring Tool out of a total number of women screened (gestational diabetes “at-risk” prevalence)	Screening records in the IDB2B database
**Adoption**			
	Site and individual level willingness to initiate B2B&Me	Site adoption: Number of site training sessions delivered Attitudes and engagement assessment of key informants and general staff	IDB2B database Key informant interviews and general staff questionnaires
		Health coach adoption: Number of health coach training sessions delivered % retention of health coaches	Liva Healthcare platform data and IDB2B database
**Implementation**			
	Fidelity of B2B&Me to protocol and uptake of the intervention	Health coaching fidelity: Health coaching fidelity index scores for each health coach % women who receive a minimum number of health coaching sessions Health coach training testing scoring % women who record goals as part of health coaching Maintenance of health behavior change goals achieved	Liva Healthcare app and platform analytics
		Program fidelity: % scheduled visits completed Number of protocol adaptations and rationale for changes	IDB2B database, exit interviews with participants and partners (where available to participate)
		Implementation challenges: Exploratory findings developed through key informant interviews, focus groups, and RCT^f^ working group minutes	IDB2B database, exit interviews with participants and partners (where available to participate)
		Health economic evaluation: Total intervention cost Integrated cost-effectiveness	Cost of intervention components Intervention effectiveness for primary outcome
**Maintenance**			
	Extent to which B2B&Me has the potential for long-term effects	Potential for future implementation across key informants and women	Semistructured interviews

^a^RE-AIM: Reach, Effectiveness, Adoption, Implementation, and Maintenance.

^b^B2B&Me: Bump2Baby and Me.

^c^IDB2B: IMPACT DIABETES Bump2Baby project.

^d^mHealth: mobile health.

^e^GDM: gestational diabetes mellitus.

^f^RCT: randomized controlled trial.

#### Reach

Reach includes data on penetration, which is how many in the service were eligible and how many of those engaged and were recruited. Participation relates to adherence and dropout. The reach measures will be collected from the absolute screening numbers for each site and recording reasons for declining or ineligibility, participation records, app analytics, and health coaching completion data. This will yield data on the proportion and representativeness of individuals who are willing to participate in the intervention. We will apply a novel trial recruitment process, whereby individuals initially express interest in participation before randomization. Once randomized, participants will be provided with an arm-specific information sheet and consent form. Thus, participants know what intervention they are signing up to and this provides some estimate of intervention reach, which is not available from a standard consenting process.

#### Effectiveness

A detailed description of the data collection for the effectiveness evaluation is available in the RCT protocol publication [29]. The maternal and child data will be collected in the IDB2B database, which is purpose built and allows for secure web-based data entry for both participants and research staff with the ability to audit record entries and changes over time. All biological samples collected will be processed on site and frozen to −80 °C before shipping to Dublin for analysis. Screening data including Monash GDM risk tool scores and medical records for GDM diagnosis will be further explored to understand how effective the GDM screening tool was in predicting GDM diagnosis.

#### Adoption

The adoption data will explore the proportion and representativeness of the setting and intervention agents willing to initiate the intervention. All quantitative data collected during the intervention will be at the site level and entered into the IDB2B database by research staff when transcription has occurred. The adoption measures will be collected using a short anonymous digital and paper attitudinal survey of hospital staff at clinical sites. All hospital staff will be invited to participate from senior management to administrative staff. The validated survey [54] asks for the staff’s opinions on screening women to provide a diet and physical activity intervention and weight management to women at high risk of developing GDM. The survey will be circulated to staff at the start and end of the project. The responses will be analyzed comparing baseline views to those collected at the end of the project to provide insight into the readiness of the inner context to uptake the intervention and whether it was normalized in the longer term. This aligns with the EPIS framework preparation and sustainment phases measurement. Additional qualitative data will be collected by conducting stakeholder interviews with maternity services staff in leadership positions to explore adoption as well as other RE-AIM aspects. Longitudinal interviews of key stakeholders such as clinical nurse managers at each clinical site will be carried out during the project, aiming for quarterly. These semistructured interviews will assess the local context and environment to measure what is happening within the hospital clinics and services that might impact the intervention and how well it fits with the current routine care.

#### Implementation

Data collected on implementation fidelity measures will come from health coach training sessions, health coaching analytics collected within the app, attendance records for meetings, and participant visits and logs for all documentation changes and reasons. The health coaching training will include training on the smartphone app and coach dashboard; an introduction specific to the trial, insights into the participant journey; both synchronous and asynchronous consultation frameworks; qualitative coaching aspects; and the management of critical time points like disengagement, relapse, and medical escalation. The training will address housekeeping and content review procedures, followed by an activity checklist and 2 initial consultation role-play exercises with feedback sessions as well as dedicated time for questions and answers. After the health coach training, each coach will complete a checklist of tasks within the app and platform and complete a practice baseline synchronous call with another experienced health coach. The health coach qualitative supervision framework assists the Liva supervisor in assessing 9 key competencies across the coaching journey. The supervisor evaluates each competency, which is scored individually, to ensure that all relevant competencies are covered in a repeatable fashion. When evaluating competency, the supervisor reviews a minimum of 3 participants using the app and 5-8 if the health coach is new to the role. Of these, the supervisor reviews 4-5 videos or interventions. While carrying out an evaluation of competency, the supervisor can allocate a 0, 1, and −1 or an N/A to each competency in the supervision framework. A score of −1 is assigned when a coach is not achieving the requirements of the competency, or when the supervisor assesses that there are significant improvements to be made. A score of 0 is assigned when a coach infrequently achieves the requirements of the competency or when the supervisor assesses that there are improvements to be made. A score of 1 is assigned when a coach fulfills the requirements of the competency or when the supervisor assesses that there are minimal insignificant improvements to be done. An “N/A” score is assigned when the area in question is not relevant or not possible to assess on this occasion. In completing the framework, a total score is calculated automatically. This score is a quantitative measure to assist the supervisor. After a supervision has taken place, the supervisor delivers feedback to the coach. This score will be evaluated alongside the fidelity index that was developed for the initial synchronous coaching sessions with independent specialist input. This fidelity index identified 18 health coaching techniques that need to be delivered in the initial health coaching session. The index assesses the use of relational techniques used to build a constructive empathic working alliance between the coaches and the study participants, and specific behavior change techniques used to facilitate individuals achieving their health goals.

The health coaching analytics include data relating to the number of participants coached, coach capacity, percentage contacts delivered on schedule, retention rate, demographics, goals set by participants, and coach rating by Liva supervisor. Women and their partners will have exit interviews at the end of the project with research staff. Health coaches will participate in focus groups following the active intervention completion in all sites. The exit interviews and focus groups will be semistructured and follow topic guides with prompts to provide a deeper understanding of implementation measures. The focus group and interview topic guides were informed by the EPIS framework, predominantly the implementation and sustainment aspects. The interviews and focus groups will be conducted by an experienced qualitative researcher independent of the maternity service. The recording of interviews and focus groups will be conducted using a digital audio recorder. The transcription will be conducted by trained staff, and all transcripts will be independently checked for accuracy before anonymization.

#### Maintenance

Participant-level maintenance of behavior change will be measured by goals achieved within the smartphone app. The adherence, retention, and loss to follow-up of participants in the mHealth coaching and the trial will inform future delivery. The cost information on the intervention delivery will be collected by participant questionnaires at 3 monthly intervals postpartum and from medical records for pregnancy data. The measures that will be collected from medical records include birth outcomes, length of hospital stay, admission to neonatal intensive care unit, and medications. The postpartum questionnaire will collect data on childcare use to support health behavior change and costs associated with health care professional visits. The health service’s interest in delivering the screening tool in usual care will be explored using key informant interviews at the trial completion.

### Data Analysis

#### Reach

The representativeness of the participants will be assessed within each site and barriers and enablers to participation will be explored using participant exit interviews.

#### Effectiveness

We will assess the RCT impact on the intervention’s prespecified primary and secondary outcomes [29]. The reach evaluation penetration data will support the prevalence analysis of women at higher risk of developing GDM.

#### Adoption

We will use process data to assess site and health coach adoption. Additional surveys and key informant interviews will be conducted over the course of the intervention to inform other aspects of the adoption. The interview transcripts will be analyzed inductively using reflexive thematic analysis [55] and deductively using normalization process theory [56] to explore this aspect.

#### Implementation

We will assess the fidelity of the health coaching delivery and the program. The fidelity of delivery will be assessed separately for synchronous coaching and asynchronous interactions. For the synchronous coaching, these will be digitally recorded. These recordings will then be reviewed, and each health coaching technique identified and timed. This will then be compared with the specified techniques to be used from the Health Coaching Manual and used to generate an index of congruency. For the asynchronous coaching, we will code the Health Coaching techniques used in each coaching message. This will then be used to generate a technique profile aggregated for each individual from all interactions and aggregated for each coach. We will also do an exploratory analysis of the key informant interviews and focus groups using thematic analysis to identify implementation challenges. The key informant interviews and focus groups will take a variety of key stakeholder perspectives including hospital staff, health coaches, and the participants. They are informed by the EPIS framework. These data will be complemented by the RCT working group meeting minutes, which ran monthly for the duration of the project and detailed implementation challenges from the perspective of the trial.

Cost-effectiveness analysis (CEA) is a well-founded and widely used method within health economics, where the costs and outcomes of a specific intervention are compared to an alternative (normally usual care). The CEA produces an incremental cost-effectiveness ratio (ICER). This ratio is compared to established willingness to pay thresholds and to similar interventions to evaluate the cost-effectiveness and comparative effectiveness of the studied intervention. The CEA explained in this analysis plan will be used to evaluate the costs and health outcomes of the IDB2B intervention compared to usual care. The results from the analysis will serve as a foundation for health care policy makers in the decision-making surrounding the implementation of the intervention.

The 2 main health outcomes for the economic evaluation are health-related quality of life measured using the quality-adjusted life year (QALY) approach and BMI 12-month postpartum. QALYs are a summary health outcome measure routinely used for economic evaluation integrating quantity or time and quality of life into a single index. QALYs will be calculated based on responses from the EuroQol 5 dimensions long (EQ-5D-L) questionnaire, and country-specific value sets will be applied. Effects (QALYs and BMI) for the economic evaluation will be measured at the individual level over an observation period corresponding to the trial follow-up time of individual patients. The cost-effectiveness analyses will be performed from both a health sector perspective and a societal perspective. Cost categories for the economic evaluation from a health sector perspective include the value of all health care consumption, pharmaceuticals, and the cost of the intervention itself. The CEA from a societal perspective entails incorporating indirect costs, that is, productivity losses due to short-term and long-term sickness (sick leave). Information on the usage of health services will be collected at the individual level for all patients enrolled in the trial. These services will be captured from the electronic case report forms maintained by IDB2B clinical sites. If a patient visits health providers that are not one of the trial centers, the number of diagnostic and treatment procedures will be obtained based on self-reporting by patients. Valuation of the resources identified and measured will be done using country-specific diagnosis-related group tariffs. Comparing treatment effects and costs between 2 alternatives results in an ICER, which is calculated as follows:



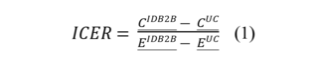



where *C^IDB2B^* and *C^UC^* are the mean costs among patients in the IDB2B and usual care arms, while *E^DB2B^* and *E^UC^* are the mean effects (QALYs or BMI).

Depending on the effect measure chosen, the ICER expresses the additional costs per unit decrease in BMI or per QALY gained if IDB2B is used by pregnant women at risk of GDM. Concluding whether the intervention or the comparator is the cost-effective alternative depends on the societal willingness to pay (for a specific country), that is, if the ICER is below the willingness-to-pay threshold the intervention will be regarded as cost-effective. The evaluation will follow the Consolidated Health Economic Evaluation Reporting Standards (CHEERS). The analysis will pool data from all countries as well as conduct country-specific estimates with a comparative analysis of the health system and contextual factors affecting cost-effectiveness across countries. To estimate the long-term cost-effectiveness of the IDB2B intervention, a Markov cohort model will be used.

#### Maintenance

The future potential implementation of the B2B&Me program will be explored through semistructured interviews with key informants and participants from each participating site. The interview transcripts will be analyzed inductively using reflexive thematic analysis [55] and deductively using normalization process theory [56] to explore this aspect. The impact of the lengthy health coaching duration will also be explored as the 18 months of coaching is novel within this field.

### Ethics Approval

The National Maternity Hospital Human Research and Ethics Committee was the primary approval site (EC18.2020) with approvals from University College Dublin HREC-Sciences (LS-E-20-150-OReilly), Junta de Andalucía Comités de Ética de la Investigación con Medicamentos/Comité Ético de Investigación Provincial de Granada (2087-M1-22), Monash Health Human Research Ethics Committee (RES-20-0000-892A), and NHS Health Research Authority and Health and Care Research Wales (21/WA/0022).

## Results

Recruitment commenced on February 9, 2021, and data collection is currently ongoing. Publication of the results is expected during 2024.

## Discussion

At the end of this 5-year project, we aim to assess the effectiveness and implementation of a complex intervention aligned with maternity services for women at high risk of developing GDM. It is clear that structured diet and physical activity interventions in antenatal care are associated with improved maternal outcomes, such as less GWG [19] and T2DM prevention, and that the capacity to implement such interventions in antenatal care settings is limited. There is a great need to conduct more pragmatic implementation research to generate learnings that could benefit the broader antenatal and postnatal care settings [18,19] and develop interventions that transcend pregnancy into the longer postpartum period in at-risk groups. The communication gap between maternity service and primary care is a well-known challenge for GDM management [22] but also wider continuity of care.

While the evaluation of the B2B&Me’s mHealth coaching intervention effectiveness will be valuable, the implementation process assessment will make an important contribution as well. The duration of the health coaching particularly through the mHealth platform is novel, with the average duration being 6 months [57]. The type 2 hybrid effectiveness-implementation study design was selected because both aspects are complementary for evaluating this complex implementation project. The effectiveness evaluation takes a best-practice approach using an RCT. An important future implementation consideration of the design was the decision to ensure the data required to screen for eligibility were collected within routine care at the first antenatal visit for all 4 countries. This will facilitate the screening being potentially embedded within usual care as opposed to other risk factor screening tools that use biochemical or nonroutine tests [58,59]. The RE-AIM framework evaluation will yield valuable data that are important for the adoption of the intervention, if proven to be efficacious, and support wider implementation learnings.

IDB2B is not without limitations. The use of an RCT design brings with it the known issues with recruitment of those with potentially different demographics to the wider maternity population and the process of seeking consent will inherently curtain reach. We will measure the penetration of the study population as a means of looking further into this potential limitation, but we will likely not be able to control it. In the analysis of the implementation, it is likely that variation across countries will be a factor that will affect our data. We are planning to measure and potentially account for socioeconomic and ethnic variability among the women participating, but we cannot account for these factors among other stakeholders. This study cannot account for changes in the standard of care provided in primary care for reasons such as the COVID-19 pandemic. Any changes that occurred within the 4 maternity services’ usual care should be accounted for within the longitudinal stakeholder interviews.

The project has the potential to provide a high-quality implementation assessment of a complex mHealth coaching intervention across multiple maternity services and the postpartum period. The IDB2B project has the potential for more general usage as an mHealth coaching platform beyond the B2B&Me RCT in the control and prevention of GDM and the long-term consequences for the mother and the offspring, if found to be effective. We expect that the results of this evaluation will yield important findings that will potentially support the improved health of women and children over the first 1000 days and beyond.

## Appendix

Multimedia Appendix 1 Grant peer review report IMPACT DIABETES B2B_ESR

## Abbreviations

B2B&Me: Bump 2 Baby and Me
CEA: cost-effectiveness analysis
CHEERS: Consolidated Health Economic Evaluation Reporting Standards
EPIS: exploration, preparation, implementation, and sustainment framework
EQ-5D-L: EuroQol 5 dimensions long
GDM: gestational diabetes
GWG: gestational weight gain
ICER: incremental cost-effectiveness ratio
IDB2B: Impact Diabetes Bump2Baby
mHealth: mobile health
QALY: quality-adjusted life year
RCT: randomized controlled trial
RE-AIM: Reach, Effectiveness, Adoption, Implementation Fidelity, and Maintenance framework
T2DM: type 2 diabetes

## Data Availability

Data can be made available upon reasonable request to the corresponding author.
