# Supplementary material for: A Complex mHealth Coaching Intervention to Prevent Overweight, Obesity, and Diabetes in High-Risk Women in Antenatal Care: Protocol for a Hybrid Type 2 Effectiveness-Implementation Study
Source: JMIR Res Protoc. 2023 Sep 18;12:e51431. doi: 10.2196/51431 (PMC10546269; doi:10.2196/51431)
Supplement: Multimedia Appendix 1 [file resprot_v12i1e51431_app1.pdf]

Associated with document Ref. Ares(2019)4951190 - 29/07/2019

Proposal Evaluation Form

EUROPEAN COMMISSION

Horizon 2020 - Research and Innovation Framework Programme

Evaluation Summary Report - Research and innovation actions/Innovation actions

**Call:**	H2020-SC1-2019-Two-Stage-RTD
**Type of action:**	RIA
**Proposal number:**	847984-2
**Proposal acronym:**	IMPACT DIABETES B2B
**Duration (months):**	60
**Proposal title:**	Implementation Action to prevent Diabetes from Bump 2 Baby (IMPACT DIABETES B2B): a low-resource system of care intervention for appropriate gestational weight gain and improved postnatal outcomes
**Activity:**	SC1-BHC-19-2019

N.	Proposer name	Country	Total Cost	%	Grant Requested	%
1	UNIVERSITY COLLEGE DUBLIN, NATIONAL UNIVERSITY OF IRELAND, DUBLIN	IE	1,459,155	33.79%	1,459,155	36.48%
2	DEAKIN UNIVERSITY	AU	43,025	1.00%	0	0.00%
3	KOBENHAVNS UNIVERSITET	DK	387,393.75	8.97%	387,393	9.68%
4	UNIVERSITY OF BRISTOL	UK	451,815	10.46%	451,815	11.30%
5	MONASH UNIVERSITY	AU	275,771.25	6.39%	0	0.00%
6	Liva Healthcare a/s	DK	696,917.5	16.14%	696,917	17.42%
7	UNIVERSIDAD DE GRANADA	ES	483,016.25	11.18%	483,016	12.08%
8	BETA TECHNOLOGY LTD	UK	451,041.25	10.44%	451,041	11.28%
9	AARHUS UNIVERSITET	DK	70,612.5	1.64%	70,612	1.77%
	Total:		4,318,747.5		3,999,949	

Abstract:
IMPACT DIABETES B2B will demonstrate the real-world implementation of an evidence-based, low-resource system-level intervention for healthy gestational weight gain and early prevention of maternal and child diabetes, overweight and obesity when delivered ‘at scale’ across antenatal settings. Gestational diabetes affects up to 18% of pregnancies worldwide and is an increasing health problem for both mothers and babies. By identifying those most at risk of developing gestational diabetes and working with them through personalised health coaching delivered via smartphone App, this project will engage, motivate and empower this target group to lead healthier lives for improved wellbeing and pregnancy outcomes. This project will demonstrate implementation within 3 European countries and Australia with clear line-of-sight on future scale-up across different contexts and resource settings via its innovative implementation toolkit and workshops for dissemination and exploitation. Pregnancy is a unique time in life with potential to influence maternal health, and the health of the next generation.
IMPACT DIABETES B2B delivers breakthrough research in nutrition, exercise and behaviour change leading to an implementable low-resource system-level intervention. The personalised feedback will empower women to manage their health, delivering cost-effective management of excess gestational and post-pregnancy weight gain to: improve pregnancy and postpartum outcomes; improve utilisation of healthcare services; and encourage ongoing maternal health with sustainable impact for mother and family. Expertise in implementation science, lifestyle change, health psychology, mHealth technology, health economics and health service delivery will lead this mixed methods project. The project will co-design the intervention system, with end-user context and management explored throughout. The work will evaluate clinical, economic and implementation outcomes to inform future practice and policy.

Evaluation Summary Report

Evaluation Result

Total score: 12.50 (Threshold: 12)

Form information

SCORING

Scores must be in the range 0-5.

Interpretation of the score:

*0 The **proposal fails to address the criterion** or cannot be assessed due to missing or incomplete information.*

***1 Poor.** The criterion is inadequately addressed, or there are serious inherent weaknesses.*

***2 Fair.** The proposal broadly addresses the criterion, but there are significant weaknesses.*

***3 Good.** The proposal addresses the criterion well, but a number of shortcomings are present.*

***4 Very good.** The proposal addresses the criterion very well, but a small number of shortcomings are present.*

***5 Excellent.** The proposal successfully addresses all relevant aspects of the criterion.Any shortcomings are minor.*

Criterion 1 - Excellence

Score: **4.00** (Threshold: 4/5.00 , Weight: -)

The following aspects will be taken into account, to the extent that the proposed work corresponds to the topic description in the work programme:
Clarity and pertinence of the objectives
Soundness of the concept, and credibility of the proposed methodology
Extent that proposed work is beyond the state of the art, and demonstrates innovation potential (e.g. ground-breaking objectives, novel concepts and approaches, new products, services or business and organisational models)
Appropriate consideration of interdisciplinary approaches and, where relevant, use of stakeholder knowledge and gender dimension in research and innovation content

The proposal addresses the criterion very well, but with a small number of shortcomings.
The proposal is in line with the call and is important for the improvement of maternal and child health. The objectives of the proposal are well-defined. The concept is sound, novel and practical. The proposed methodology is strong. There is a clear interdisciplinary approach, with a solid evaluation plan. All cross-cutting priorities are generally very well addressed. All intervention components are previously tested and the risk screening tool has been validated. The different intervention components build on already available evidence and go beyond the state of the art.
Nevertheless, the proposal did not provide sufficient details on the potential role that men/partners might have in the decision-making process related to pregnancy. It was unclear whether local cultures and gender dynamics would be explored as a potential barrier or facilitator. There was a lack of details on and rationale for the cost-effectiveness analysis, given the lack of clear hypotheses on savings and QALY gains.

Criterion 2 - Impact

Score: **4.00** (Threshold: 4/5.00 , Weight: -)

The following aspects will be taken into account:
The extent to which the outputs of the project would contribute to each of the expected impacts mentioned in the work programme under the relevant topic
Any substantial impacts not mentioned in the work programme, that would enhance innovation capacity, create new market opportunities, strengthen competitiveness and growth of companies, address issues related to climate change or the environment, or bring other important benefits for society
Quality of the proposed measures to:
- exploit and disseminate the project results (including management of IPR), and to manage research data where relevant
- communicate the project activities to different target audiences

The proposal addresses the criterion very well, although certain improvements are still possible.
The expected impacts are generally well addressed. Overall, the proposal addresses effectively the knowledge-do gap of interventions for decreasing Body Mass Index (BMI) in pregnant and postpartum women up to one year. The proposed approach is considered innovative. The focus across the 1000 days is permitting a continuity of care from pregnancy into the postpartum period. The project will provide a good understanding of what works and what does not when implementing the intervention. The engagement strategy and segmentation of the target key audiences were clearly described. It directly contributes to the achievement of SDG2 and SDG3.
The proposal has a developed and credible dissemination and communication plan that reaches all target audiences (from end-users to policy makers) and includes open access, IPR management, and data management.
However, equity was not sufficiently addressed, without a specific focus on the needs of disadvantaged or vulnerable groups. Additionally to different languages, the structural factors at macro-, meso- and micro-levels such as cultural differences and different policies should have been better addressed and as such, the transferability of findings to non-High Income Countries remains insufficiently described in the proposal. The sustainability of the intervention after the project end was unclear.

Criterion 3 - Quality and efficiency of the implementation

Score: **4.50** (Threshold: 3/5.00 , Weight: -)

The following aspects will be taken into account:
Quality and effectiveness of the work plan, including extent to which the resources assigned to work packages are in line with their objectives and deliverables
Appropriateness of the management structures and procedures, including risk and innovation management
Complementarity of the participants and extent to which the consortium as a whole brings together the necessary expertise
Appropriateness of the allocation of tasks, ensuring that all participants have a valid role and adequate resources in the project to fulfil that role

The proposal addresses the criterion very well, but with small shortcomings.
The proposed implementation plan is almost excellent. The team proposed a solid, multi-level intervention with partners of proven expertise in the field. The activities to implement the proposed project are laid out clearly in nine work packages, the sequence and approach of each being well grounded in implementation research theory. The consortium as a whole has the expertise and wide experience in similar complex interventions, with a well-established distribution of leads and tasks. The roles and responsibilities of each consortium member are well-articulated across these work packages. Gender balance and management structure are appropriate and sufficiently described.
For the allocation of roles and resources, the estimated person months for WP 2 may not be enough to develop the main technology platform (App and Health Coaching Library) needed for the proposed project.

Scope of the proposal

Status: **Yes**

Comments (in case the proposal is out of scope)

Not provided

Operational Capacity

Status: **Operational Capacity: Yes**

If No, please list the concerned partner(s), the reasons for the rejection, and the requested amount.

Not provided

Associated with document Ref. Ares(2019)4951190 - 29/07/2019

Exceptional funding of third country participants/international organisations

A third country participant/international organisation not listed in General Annex A to the Main Work Programme may exceptionally receive funding if their participation is essential for carrying out the project (for instance due to outstanding expertise, access to unique know-how, access to research infrastructure, access to particular geographical environments, possibility to involve key partners in emerging markets, access to data, etc.). (For more information, see the Online Manual)

Based on the information provided in the proposal, we consider that the following participant(s)/international organisation(s) that requested funding should exceptionally be funded:
(Please list the Name and acronym of the applicant, Reasons for exceptional funding and the Requested grant amount.)

Not provided

Based on the information provided in the proposal, we consider that the following participant(s)/international organisation(s) that requested funding should NOT be funded:
(Please list the Name and acronym of the applicant, Reasons for exceptional funding and the Requested grant amount.)

Not provided

Use of human embryonic stem cells (hESC)

Status: **No**

If yes, please state whether the use of hESC is, or is not, in your opinion, necessary to achieve the scientific objectives of the proposal and the reasons why. Alternatively, please state if it cannot be assessed whether the use of hESC is necessary or not because of a lack of information.

Not provided

Overall comments

Not provided

European Commission

FUNDING & TENDERS PORTAL DIGITAL SEAL

Digitally sealed by the European Commission
Date: 2019.07.29 15:12:11 CEST

This document is digitally sealed. The digital sealing mechanism uniquely binds the document to the modules of the Funding & Tenders Portal of the European Commission, to the transaction for which it was generated and ensures its integrity and authenticity.

Any attempt to modify the content will lead to a breach of the electronic seal, which can be verified at any time by clicking on the digital seal validation symbol.
